# A multimodal clinical data resource for personalized risk assessment of sudden unexpected death in epilepsy

**DOI:** 10.3389/fdata.2022.965715

**Published:** 2022-08-17

**Authors:** Xiaojin Li, Shiqiang Tao, Samden D. Lhatoo, Licong Cui, Yan Huang, Johnson P. Hampson, Guo-Qiang Zhang

**Affiliations:** ^1^Department of Neurology, The University of Texas Health Science Center at Houston, Houston, TX, United States; ^2^Texas Institute for Restorative Neurotechnologies, The University of Texas Health Science Center at Houston, Houston, TX, United States; ^3^School of Biomedical Informatics, The University of Texas Health Science Center at Houston, Houston, TX, United States

**Keywords:** epilepsy, sudden unexpected death in epilepsy (SUDEP), multimodal clinical data resource, ontology-driven system design, personalized risk assessment, machine learning, deep learning

## Abstract

Epilepsy affects ~2–3 million individuals in the United States, a third of whom have uncontrolled seizures. Sudden unexpected death in epilepsy (SUDEP) is a catastrophic and fatal complication of poorly controlled epilepsy and is the primary cause of mortality in such patients. Despite its huge public health impact, with a ~1/1,000 incidence rate in persons with epilepsy, it is an uncommon enough phenomenon to require multi-center efforts for well-powered studies. We developed the Multimodal SUDEP Data Resource (MSDR), a comprehensive system for sharing multimodal epilepsy data in the NIH funded Center for SUDEP Research. The MSDR aims at accelerating research to address critical questions about personalized risk assessment of SUDEP. We used a metadata-guided approach, with a set of common epilepsy-specific terms enforcing uniform semantic interpretation of data elements across three main components: (1) multi-site annotated datasets; (2) user interfaces for capturing, managing, and accessing data; and (3) computational approaches for the analysis of multimodal clinical data. We incorporated the process for managing dataset-specific data use agreements, evidence of Institutional Review Board review, and the corresponding access control in the MSDR web portal. The metadata-guided approach facilitates structural and semantic interoperability, ultimately leading to enhanced data reusability and scientific rigor. MSDR prospectively integrated and curated epilepsy patient data from seven institutions, and it currently contains data on 2,739 subjects and 10,685 multimodal clinical data files with different data formats. In total, 55 users registered in the current MSDR data repository, and 6 projects have been funded to apply MSDR in epilepsy research, including three R01 projects and three R21 projects.

## 1. Introduction

Epilepsy is characterized by unpredictable seizures that occur recurrently and spontaneously (Fisher et al., 2014). Seizures affects approximately one in every twenty-six adults in the United States in their lifetime (Hesdorffer et al., 2011). In an epileptic seizure, large numbers of brain neurons are involved in an excessive, synchronized, and inappropriate electrical discharge that triggers signs and symptoms (Goldenberg, 2010). These result in a large variety of signs and symptoms depending on the brain regions involved (Clark and Kruse, 1990). Approximately one-third of epilepsy patients are unable to become seizure-free with currently available treatments, increasing their risk of sudden unexpected death in epilepsy (SUDEP; Petrucci et al., 2021).

SUDEP is a catastrophic and fatal complication of epilepsy and is the primary cause of mortality in those who have uncontrolled seizures (Devinsky et al., 2016a). It ranks second only to stroke in terms of years of potential life lost due to neurological disease (Thurman et al., 2014). Epilepsy patients who die from SUDEP have no obvious cause or mechanism of death that can be identified at autopsy (Okanari et al., 2020). In epilepsy clinic populations, the incidence of SUDEP ranges between 1.1 and 2.9 per 1,000 patient-years, whereas it is 6.3–9.3 per 1,000 patients with intractable epilepsy, posing a significant public health concern (Zhao et al., 2021). While several multifactorial processes have been involved including cardiac (Devinsky et al., 2016a), respiratory (Lacuey et al., 2018, 2019; Vilella et al., 2019b, 2021), autonomic dysfunction leading to arrhythmia, hypoxia, and cessation of cerebral and brainstem function, the mechanisms underlying SUDEP are not completely understood (Okanari et al., 2020; Petrucci et al., 2021).

A 2010 report by the Institute of Medicine (IOM), “Elements of a National Strategy for Accelerating Research and Product Development for Rare Diseases,” suggests a national strategy that uses scarce funding, expertise, data, and biological specimens efficiently and effectively by sharing research resources and infrastructure (Zhang et al., 2014). This recommendation is especially relevant to SUDEP research due to its relatively low rate of reported incidences (Devinsky, 2011; Devinsky et al., 2016b, 2018). Therefore, multiple epilepsy monitoring units (EMUs) could collaborate effectively, sharing data to build a larger cohort of potential SUDEP patients by using state-of-the-art informatics and data analytics infrastructure (Zhang et al., 2014).

In this paper, we describe the design and development of the Multimodal SUDEP Data Resource (MSDR) by the Informatics and Data Analytics Core (IDAC) of the Center for SUDEP Research (CSR). MSDR is a system for the structural and semantic harmonization of and web-based access to multimodal clinical data, which is captured and uploaded from multiple individual institutions and processed at the central data repository. Data processing tasks include data integration, data curation, and data conversion (Sahoo et al., 2012; Zhang et al., 2014; Tao et al., 2022). It provides a single point of access to analysis-ready multimodal clinical data to facilitate SUDEP research.

## 2. Background

**Figure 1:**
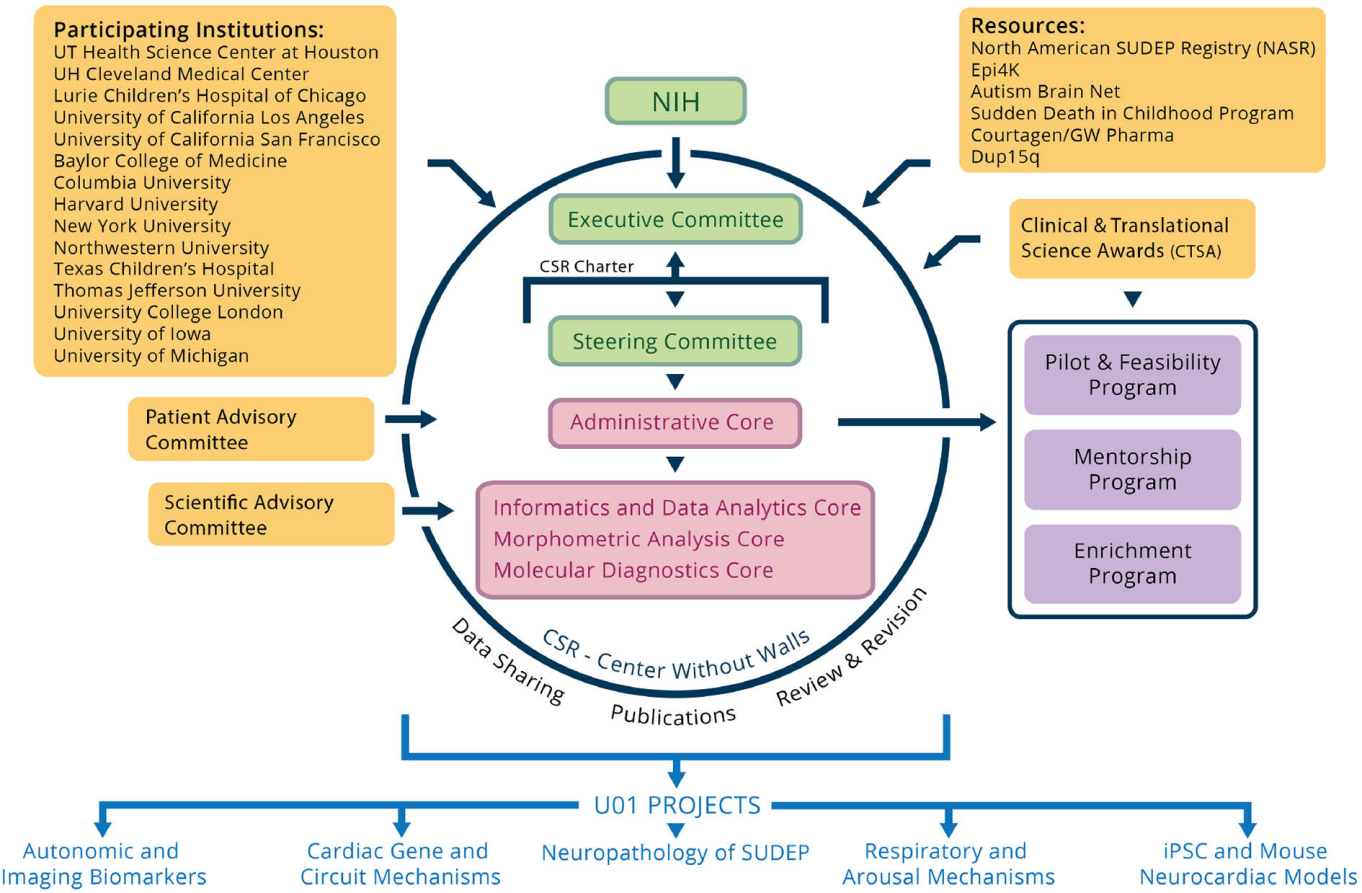
Overview of the program components of the Center for SUDEP Research.

The overview of CSR is shown in Figure 1. CSR is a multi-site cross-disciplinary collaboration composed of researchers from 15 institutions across the United States and Europe to understand SUDEP. This investment by National Institute of Neurological Disorders and Stroke (NINDS) over nearly 5 years promises to catalyze research on SUDEP and dramatically enhance our understanding of this devastating phenomenon. The participating institutions of CSR includes Baylor College of Medicine, University Hospitals Cleveland Medical Center (UH), Lurie Children's Hospital of Chicago, Columbia University, Harvard University, New York University (NYU), Northwestern University (NW), Texas Children's Hospital, Thomas Jefferson University (TJU), University of California, Los Angeles (UCLA), University of California, San Francisco (UCSF), University College London (UCL), University of Iowa (UIowa), University of Michigan, and The University of Texas Health Science Center at Houston. CSR collects seven types of clinical data for analysis including patient reports from EMUs, electroencephalography (EEG) signal data, imaging data, bio-chemistry data, DNA data, follow-up forms, and SUDEP forms. UH, NYU, NW, UCLA, UCL, TJU, and UIowa are the seven CSR institutions that contribute clinical data to the central data repository.

The goal of CSR is to support the SUDEP research community to maximize the value of multimodal clinical data, tools, best practices, and other resources from the CSR in pursuit of the overall goal of understanding risk factors and brain mechanisms of SUDEP (CSR, 2022). CSR governance is carried out through partnership with National Institutes of Health (NIH)/NINDS and is comprised of an Executive Committee and a Steering Committee. The Steering Committee consists of the corresponding Principal Investigator from each Core/Project, the Directors, and the NINDS Scientific Program Officer. The Steering Committee has primary responsibility for the establishment of priorities, development of common protocols, and review of progress for the center. The Executive Committee consists of eight voting members: the CSR Co-Directors, NINDS Scientific Program Officer, NINDS Administrative Program Officer, two additional NIH staff, and two rotating members (one basic science, one clinical) from the U01 corresponding PIs.

CSR is comprised of the following components: (1) the Administrative Core, which coordinate interactions between core and scientific programs, assure compliance with regulatory approvals and safety protocols, assess and review the quality and efficiency of the cores and projects, and monitor overall budget and annual reallocation; (2) the Informatics and Data Analytics Core, which aims to build on the progress already achieved through a specific infrastructure and to expand and broaden the sharing and utilization of research resources among CSR partners; (3) the Morphometrics Analysis Core, responsible for developing a repository of clinical imaging studies of SUDEP cases enrolled in the center; (4) the Molecular Diagnostic Core, which provides DNA sequencing and analysis of samples from individuals with epilepsy who have died prematurely or who have a high clinical risk of SUDEP; and (5) the five scientific U01 projects, which focus on different aspects of SUDEP research.

## 3. Approach

**Figure 2:**
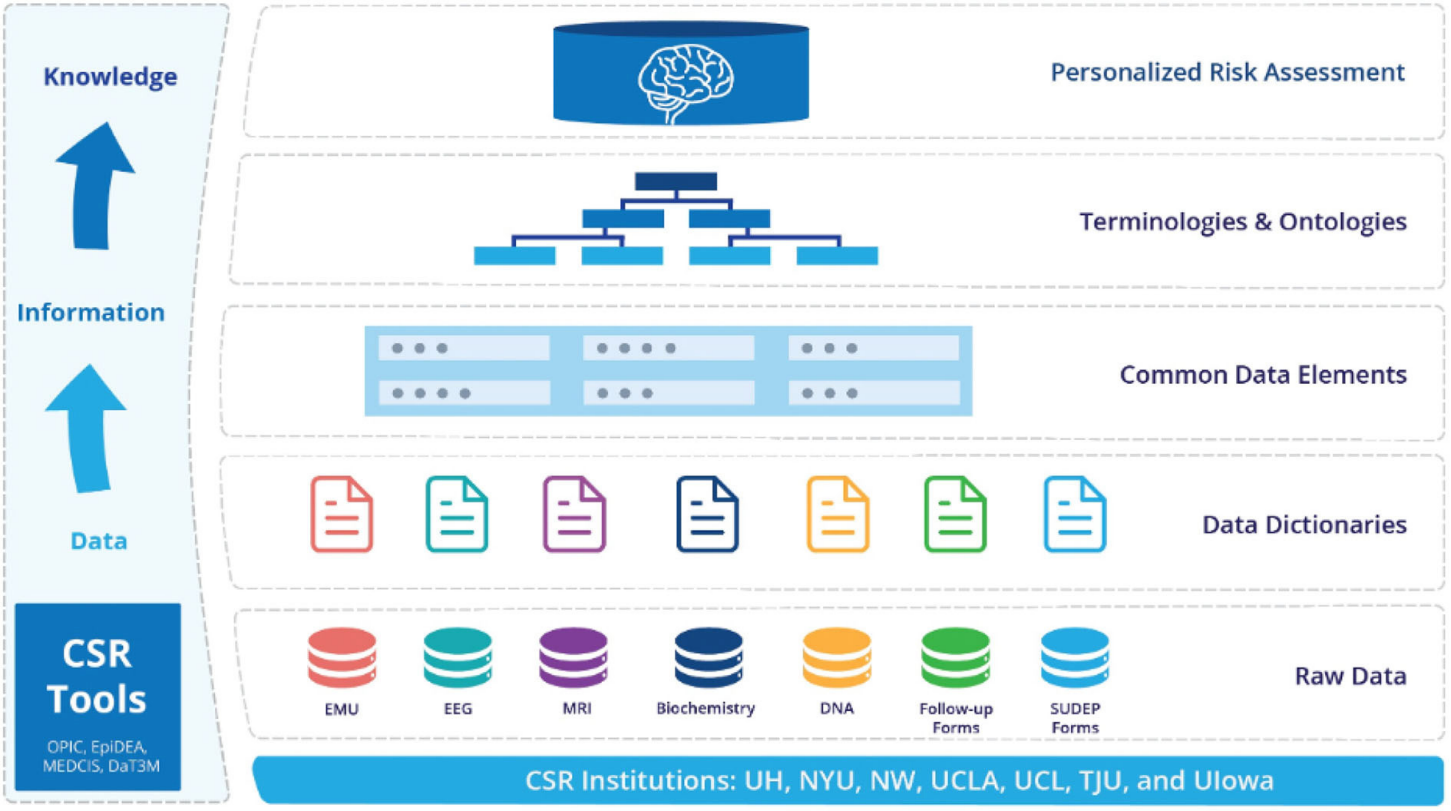
Overall data ecosystem strategy for MSDR.

MSDR is guided by the idea that universally applicable computational methodology and principles (Wing, 2006, 2008) should be incorporated systematically in managing multimodal clinical data. Figure 2 shows the overall data strategy of MSDR. We recognize that raw data, data dictionaries, common data elements, controlled terminologies, and ontologies are a cascading chain of digital resources of progressively higher conceptual degrees (Figure 2) along the data-information-knowledge-wisdom (DIKW) hierarchy (Frické, 2019). The DIKW hierarchy (other than wisdom) can be considered as data; there is value added in the direction of findable, accessible, interoperable, and reusable (FAIR; Wilkinson et al., 2016) when we link consecutive data entities in this cascading chain through analytics, annotation, and mapping. This strategy is expected to offer opportunities for new interfaces (for human or for machine) for data interpretability and integrability, leading to enhanced rigor and reproducibility.

### 3.1. Data modeling

**Figure 3:**
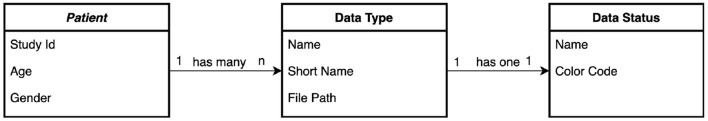
Three core data models in MSDR.

MSDR includes three core data models: Patient, Data Type, and Data Status. As depicted in Figure 3, one patient can have many data types while each data type has one data status (i.e., available or not). In the current MSDR, seven types of data: EMU reports, EEG signal data, MRI imaging data, biochemistry data, DNA data, follow-up forms, and SUDEP forms. EMU report contains clinical information generated during patient's stay in the epilepsy monitoring units including medications, seizure events, EEG findings, seizure classifications, etc. EEG signal data are in European Data Format (EDF) and well-annotated with epilepsy related events. MRI data consists of research grade MRI or CT files. Biochemistry data captures patients' blood sample-related information. DNA data record what gene tests are done for patients. Annual follow-ups with patients were performed in the CSR study, and the generated information is captured in follow-up forms. SUDEP forms capture essential information for patients who are confirmed died of SUDEP events. Different types of data are linked by the patient's unique CSR study ID (Zhang et al., 2015).

### 3.2. Epilepsy metadata: Common data elements and provenance information

The MSDR uses a metadata-guided approach to achieve uniform semantic interpretation of data elements across the entire spectrum of data integration activities: for annotating source data, for interfaces to query and search data, and for tools that access and assist in analysis.

Existing terminological systems do not cover the epilepsy domain in sufficient detail to meet the goals of the CSR. For this reason, our metadata-guided approach involves the Epilepsy and Seizure Ontology (EpSO; Sahoo et al., 2014), which models the necessary domain concepts to describe epilepsy phenotype data at significant level of detail by following an established four-dimensional classification framework in epilepsy. EpSO covers concepts of seizures, location of seizures, etiology, and related medical conditions according to the four-dimensional scheme. In addition, it models EEG patterns and comprehensive drug information (anti-epileptic, neuroleptic, and anti-depressants) by using the U.S. National Library of Medicine RxNorm standard. EpSO consists of over 1,300 concepts and integrates the latest International League Against Epilepsy (ILAE) recommendations, and the concepts in EpSO are mapped to the NINDS Common Data Elements (CDE), which represents nine categories of terms describing imaging, neurological exam, neuropsychology, seizures, and syndromes. EpSO has been successfully used to streamline data capture and integration processes and user interfaces, and to enable mapping across distributed databases to support federated queries, as well as to support centralized data curation while new data is continuously generated and integrated from multiple sites.

### 3.3. Functional components of data ecosystem in MSDR

**Figure 4:**
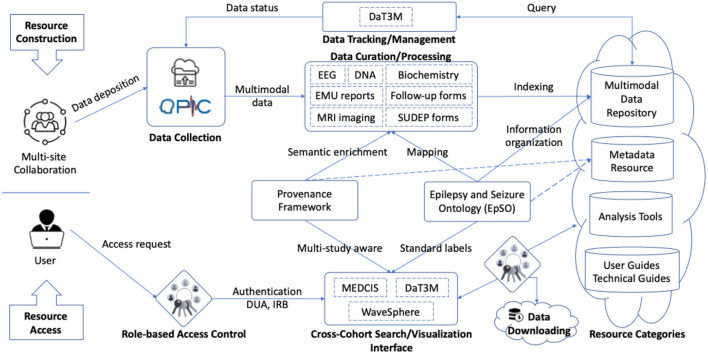
Functional components representing the connections and interactions in MSDR data ecosystem. Seven CSR institutions that contribute clinical data to the central data repository: UH, NYU, NW, UCLA, UCL, TJU, and UIowa. EpSO plays a central role in coordinating and facilitating incremental resource construction **(top)** and resource access **(bottom)**.

Our design involves a set of MSDR data ecosystem functional components to flexibly accommodate the deposition of a growing set of new tools and data. The MSDR data ecosystem consists of two main parts: Resource Construction and Resource Access (as shown in Figure 4). Resource Construction allows seven different types of data from individual institution to be curated, mapped, and integrated into MSDR on a cohort-by-cohort basis over time.

Our strategy of curation includes two-stage process to curate multimodal clinical data from different individual institutions and make them publicly available. In stage one, our team performs the curation of datasets deposited from each individual CSR institution. All data files from each institution are transformed into a standard common format using an automated script. For example, the original raw EEG signal data are in different formats depending on the EEG monitoring devices used at each institution. The script systematically converts the raw EEG signals into European Data Format (EDF)—a standard file format designed for exchange and storage of medical time series. In stage two, the curated files are indexed by institution names, patient study IDs and data types. The statuses of data completeness are also systematically tracked. In general, there are four categories of incremental available resources, including: (1) multimodal clinical data repository; (2) metadata for epilepsy research; (3) tools for data analytics, cross-cohort exploration, and visualization; and (4) user guides, technical guides, and study documents originated from individual institution.

A number of tools are utilized in Resource Construction and Resource Access of MSDR, including:

- The Ontology-driven Patient Information Capture (OPIC) system (Sahoo et al., 2012), leveraging EpSO, provides a flexible web-based interface to capture data describing demography, patient history, details of paroxysmal events, medication, results of prior electrophysiological evaluations, and patient diagnosis (as shown in the *Data Collection* component in Figure 4).
- Epilepsy Data Extraction and Annotation (EpiDEA), which is an ontology-driven clinical free text processing system that extends the clinical Text Analysis and Knowledge Extraction System (cTAKES; Savova et al., 2010) for analyzing epilepsy-specific clinical reports (applied to the *Data Curation/Processing* component in Figure 4).
- Multi-Modality Epilepsy Data Capture and Integration System (MEDCIS; Zhang et al., 2014), which is a multi-modality data capture and Integration system for epilepsy data integration across multiple EMUs with both retrospective and prospective patient information (as shown in the *Cross-Cohort Search/Visualization Interface* component in Figure 4). MEDCIS is adopted as the informatics and data infrastructure hosting MSDR, and the MEDCIS cross cohort query interface is deployed as the web portal for MSDR (MEDCIS, 2022).
- Data Tracker for Multi-faceted Management of Multi-site Clinical Research Data (DaT3M; Tao et al., 2022), which is piloted to support the data management for MSDR (as shown in the *Data Tracking/Management* component in Figure 4).
- Data Slice Downloader (Tao, 2021), which is a software package supporting batch-downloading of CSR patient cohort (applied to the *Data Downloading* component in Figure 4). It simplifies the downloading of large source files by users with appropriate credentials of data access and use agreements and IRB reviews, which are also tracked and managed through the MSDR web portal.
- Interactive visualization system for physiological signal recording, named WaveSphere (Li, 2019), which queries and interactively renders physiological signal recording of interest and corresponding annotations (as shown in the *Cross-Cohort Search/Visualization Interface* component in Figure 4).

### 3.4. Risk marker extraction

MSDR provides a comprehensive, curated prospectively constructed repository of epilepsy-related data consisting of electrophysiological signals linked to risk factors and outcome data for over 2,700 epilepsy patients (with a broad spectrum of age, social, racial, and ethnic) with thousands of 24-hour recordings. Such a rich and diverse dataset provides a solid foundation for machine learning and deep learning application development in personalized risk assessment of epileptic seizure and SUDEP. With MSDR, we utilize different techniques to extract the risk makers of SUDEP and detect/predict epilepsy-related clinical events, including rule-based Natural Language Processing (NLP) and physiological signal analysis based on machine learning and deep learning, including:

- Seizure information extraction using NLP
  Early onset of seizure is a potential risk factor for SUDEP. However, the first seizure onset information is often documented as clinical narratives in EMU discharge summaries. Manually extracting first seizure onset time from discharge summaries is time consuming and labor-intensive. Our approach adopts a rule-based NLP pipeline to automatically extract the temporal information of patients' first seizure onset from EMU discharge summaries.
- Seizure identification
  By analyzing off-line EEG signals, trained neurologists and neurophysiologists are able to identify characteristic patterns of disease, such as inter-ictal spikes and seizures, as well as disease information, such as seizure frequency, seizure type, etc. The obtained disease information is to provide support for therapeutic decisions. Manually reviewing and analyzing recordings is labor-intensive and error-prone, as it usually takes a well-trained expert several hours to analyze 1 day of recordings from one patient. These limitations have motivated researchers to develop automated techniques to recognize seizures and other electroclinical phenomena. A focus of our research is to develop an automated method to identify seizure signal segments and non-seizure segments in off-line EEG signals to assist neurologists in making a diagnosis (Yao et al., 2021).
  Our seizure identification strategy leverages a web-based signal data management and visualization system for epileptic seizure research, named SeizureBank (Li et al., 2019), which includes a dataset of analysis-ready digital signal recordings of seizures and related data from MSDR.
- Seizure forecasting and prediction
  Studying periodicities in seizure patterns has revealed that more than 90% of individuals experience circadian rhythms in their seizures, and many also experience multiday, weekly, or longer cycles (Foundation, 2016; Stirling et al., 2021). Such results provide insight into new approaches to improving seizure prediction algorithms and serve as proofs-of-concept for implementing a circadian forecasting framework (Lee, 2018). Over the past decades, seizure forecasting has seen a lot of progress, including improvements in algorithms using machine learning and exploration of other seizure susceptibility measures besides EEG, such as physiological biomarkers, behavioral changes, environmental factors, and cyclic seizure patterns (Stirling et al., 2021). Instead of projecting whether a seizure will occur or not based on a circadian cycle, seizure prediction is focused on identifying the brain state wherein there is a high probability of a seizure occurrence (Dumanis et al., 2017). Many seizure prediction approaches have been developed using machine learning-based techniques (Natu et al., 2022). However, current seizure prediction work has two limitations: (1) many public seizure datasets consist of a small number of patients, which limits patient diversity and results in unreliable performance and low generalizability to large populations; (2) most of the evaluations use segments of time with small periods, which do not show the performance under real-world situation (Huang, 2019).
  MSDR provides thousands of 24-hour richly annotated seizure-related physiological signal recordings, and those data can be retrieved, visualized, and exported with customized conditions in SeizureBank. Our seizure prediction model consists of a transfer learning framework to extract and pre-process data, train from a base model and evaluate the predictor based on MSDR.
- Post-ictal Generalized EEG Suppression (PGES) detection
  PGES is a potential EEG biomarker of SUDEP risk (Lhatoo et al., 2010; Wu et al., 2016; Vilella et al., 2019a). PGES is a period of brain inactivity after a seizure. It most often occurs after generalized tonic-clonic seizures (GTCs), particularly in those arising from sleep and is related to ictal decerebration, post-ictal immobility, lack of early oxygen administration, duration of oxygen desaturation and lower SpO2 nadir values (Alexandre et al., 2015; Kuo et al., 2016; Esmaeili et al., 2018). GTCs are the most significant risk factor for SUDEP (Wu et al., 2016). PGES is defined as diffuse EEG background attenuation (<10μ*V*) in the post-ictal period (Asadollahi et al., 2018). Prolonged PGES (>50 s) has been reported in refractory epilepsy patients who are at risk of SUDEP (Lhatoo et al., 2010). For each 1 s increase in PGES duration, the odds of SUDEP increased by a factor of 1.7% (*p* < 0.005; Lhatoo et al., 2010).
  Clinically, determination of PGES duration is manually performed by human experts through visual inspection of EEG signals. According to its definition, detection of PGES appears to be straightforward by identifying a period of low amplitude EEG signals after the seizure. However, in practice, actual data recorded in the EMUs contain high amplitude physiological artifact (e.g., breathing, muscle, and movement artifacts). Automated PGES detection tools are highly desirable to assist clinical personnel in review and annotation of PGES in EEG signals. Our model is the first time to use machine learning-based method for automatic PGES detection.

### 3.5. Hosting environment and data access

The MSDR is deployed in the data center of the University of Texas Health Science Center at Houston (UTHealth) with full security and backup support by the IT team of UTHealth (MEDCIS, 2022). The MSDR runs on a CentOS 7 Virtual Machine. The MSDR web portal, MEDCIS, is coded in Ruby on Rails 4.1 and run on Phusion Passenger for Apache web servers. The server connect to the Research File Area which grants the 120 TB of storage that can be scaled as needed as data storage requirements increase.

The MSDR offers two main levels of data access: online and offline. The MEDCIS supports online data exploration, and the DaT3M and WaveSphere tool supports online data visualization. Such online tools are implemented as web applications in the underlying architecture. For the offline access, users can use Data Slice Downloader to download the required data and perform data visualization and analysis activities with offline tools, such as EDFbrowser (Beelen, 2013) for physiological signals, on their local computational resources.

## 4. Results

### 4.1. Annotated and integrated datasets in MSDR

In May 2022, MSDR had 10,678 data components for 2,739 patients, and the total file size was over 30 terabytes (as shown in Table 1). These patients were from seven participating institutions, including 1,082 from UH, 297 from NYU, 237 from UCLA, 450 from NW, 210 from TJU, 293 from UCL, and 170 from UIowa. Different institutions had disparate patterns of data availability. For example, only UH, UCLA, and UCL contributed imaging data; and bio-chemistry data only came from UH and TJU. No site captured all modalities of data. The number of each data modality showing in Table 1 reflects the curated and available data in the MSDR. The CSR team is actively working on more data processing for sites UCLA, UCL, and NYU.

**Table 1:** Summary statistics of each data modality in MSDR.

**Center**	**No. of**	**EMU**	**EEG**	**MRI**	**Bio-chemistry**	**DNA data**	**Follow-up**	**SUDEP forms**
	**patients**	**reports**	**recordings**	**imaging**	**data**		**forms**	
UH	1,082	1,644	1,676	126	137	456	981	22
NW	450	504	505	0	0	7	296	1
NYU	297	288	308	0	0	124	283	1
UCLA	237	215	235	207	0	0	143	0
TJU	210	231	251	0	40	135	161	2
UCL	293	345	294	296	0	0	288	3
UIowa	170	171	171	0	0	0	137	2
Total	2,739	3,398	3,440	629	177	722	2,289	30

### 4.2. Tools for data collection, curation, exploration, and visualization

#### 4.2.1. The ontology-driven patient information capture system

OPIC (Sahoo et al., 2012) leveraged EpSO to automatically generate multi-level drop-down menus that were populated with only relevant terms based on previous user selection (skip patterns) and branching logic to model combinations of user selections. The OPIC system supported multiple types of data entry requirements, such as admission notes, progress notes for a patient, and discharge summary reports along with the functionality to support the input and storage of multi-modal patient data in form of EEG, EKG, and MRI reports. The OPIC forms were primarily composed of the structured data entry widgets that reduced user-generated errors, supported automated consistency checking, and ensured data completeness, using EpSO as the reference terminology system. The features of OPIC made it an ideal resource for capturing patient information and supporting multi-center epilepsy projects of similar magnitude nationwide.

#### 4.2.2. Epilepsy data extraction and annotation

EpiDEA (Cui et al., 2012, 2014) processed two types of textual content in clinical notes: the semi-structured sections with attribute-values pairs and the unstructured sections with sentence-based text. An EpSO-driven epilepsy named entity recognition module and a negation detection module processed the output of these modules. EpiDEA used EpSO as the knowledge resource for processing specialized epilepsy terms to support three functionalities: term disambiguation, term normalization, and query expansion using subsumption reasoning. The EpiDEA system also incorporated a visual interface for cohort identification that could be directly used by clinical researchers. EpiDEA was used to identify patients using constraints deciding seizure semiology, EEG and MRI patterns, and anti-epileptic drug medication, which were of particular interest in the study of SUDEP.

#### 4.2.3. Multi-modality epilepsy data capture and integration system

MEDCIS (Zhang et al., 2014) offered the following collection of main functionalities, each of which had been tested and validated independently: (1) a standardized data entry platform for patient information at different points of care; (2) an epilepsy-focused natural language processing (NLP) tool to extract patient information from clinical free text in existing patient records; (3) an integrated signal processing application for clinicians to seamlessly interface between signal data and patient information; and (4) a query environment to identify patient cohorts using data integrated from multiple sources based on a shared ontology.

**Figure 5:**
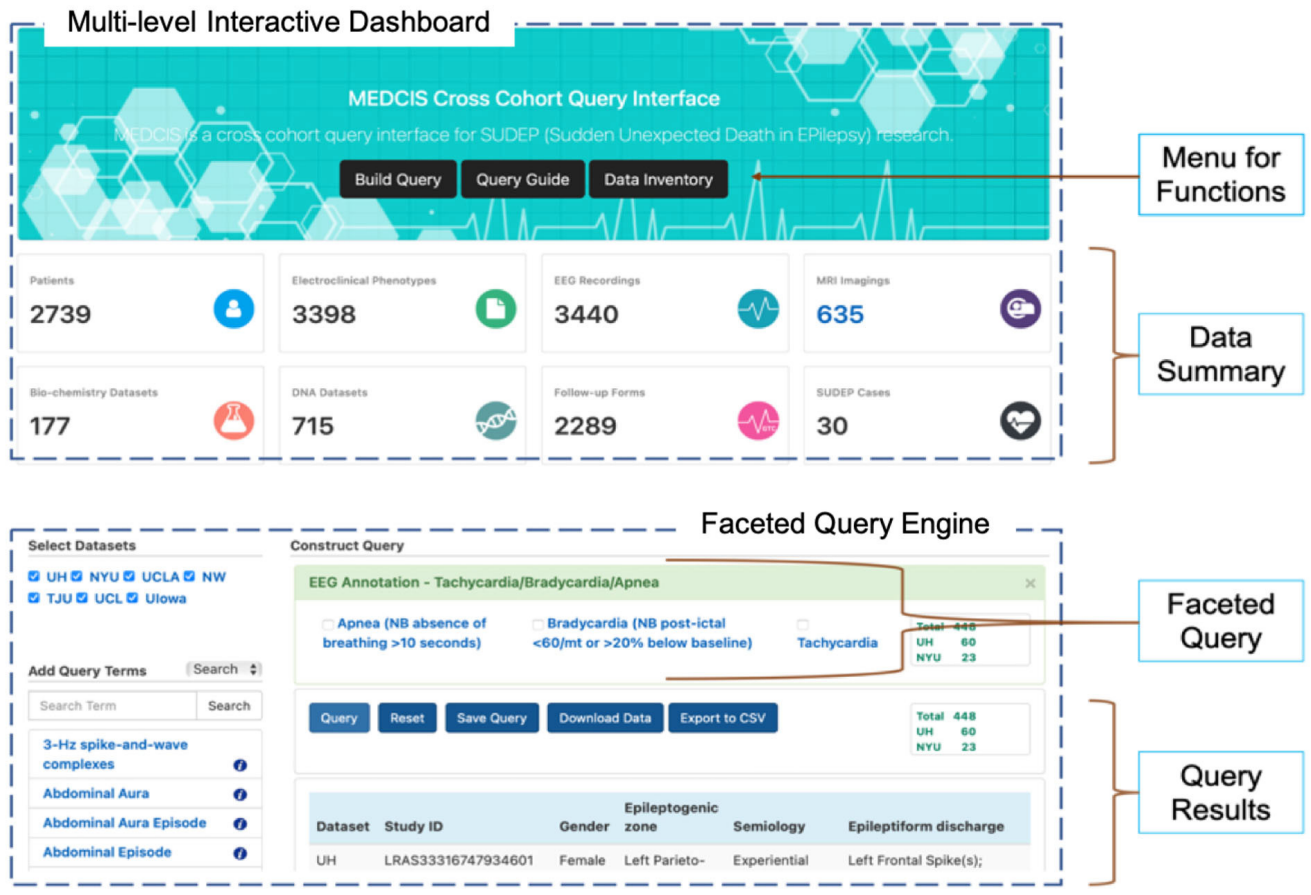
MEDCIS interface including a multi-level interactive dashboard **(top)** and a faceted query engine **(bottom)**.

The MEDCIS interface (as shown in Figure 5) included a multi-level interactive dashboard and a faceted query engine. The multi-level interactive dashboard quickly summarized the data collection. Details such as the total of complete patients, available data types in each dataset, and the total datasets of each data type were presented in the dashboard. A faceted query engine dynamically built data subsets with specific characteristics. It is critical for CSR investigators to be able to find subgroups of study data for specific purposes, including auditing for data quality and data completeness, and ensuring data interpretability and integrability.

#### 4.2.4. Data tracker for multi-faceted management of multi-site clinical research data

DaT3M (Tao et al., 2022) integrated 10,678 data components of seven different modalities for 2,739 patients from CSR's seven data contributing institutions. DaT3M provided five main features: (1) a data overview interface to provide a quick summary of the entire data collection in the central repository including information such as the total number of complete datasets, the number of datasets from each individual institution, available data types in each dataset, and the total number of datasets of each data type; (2) individual center data portal to provide a dedicated work-space for each participating institution to manage their own contributed data; (3) a data status panel for real-time status checking of multi-modal data, which was provided in institutional portal using data visualization techniques to track the statuses of all data types for each subject; (4) a data query engine to build patient cohort, which was necessary to identify the subset of patients with specific characteristics; and (5) a data slice downloader to deliver user-specified data subsets from the central repository for secondary data analysis. The features of DaT3M made it an ideal tool for supporting large-scale multi-site collaboratives.

**Figure 6:**
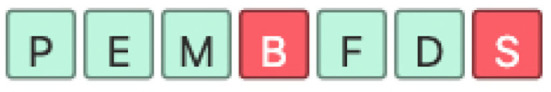
Patient data statuses of seven data types: **P**, EMU reports; **E**, EEG signal data; **M**, MRI imaging data; **B**, biochemistry data; **F**, follow-up forms; **D**, DNA data; **S**, SUDEP forms.

To provide an intuitive and concise view of patient data and their statuses, DaT3M used a colored squared box to represent each data type as well as its status. The short name of the data type was enclosed in the squared box. Green color indicated the data status was available and red denoted that the data status was missing or not available. For instance, given a specific patient, a squared box with letter “P” in green indicated that the patient's EMU reports were available. Figure 6 shows a panel of seven colored square boxes, representing the overall status of one patient dataset in CSR. The data types were evenly placed horizontally. The color of each data type (as “Color Code” referenced in Figure 3) indicated the availability status of the corresponding patient data. Therefore, the available data for the patient shown in Figure 6 included EMU reports, EEG signal data, MRI imaging data, follow-up forms, and DNA data, while biochemistry data and SUDEP form were not available.

#### 4.2.5. Data slice downloader

DaT3M integrated with MEDCIS query engine which employed concepts from EpSO to build patient cohorts. Once a patient cohort was created, DaT3M allowed end users to download the cohort data using a scalable download framework. A patient cohort often contains thousands of data files with size in terabyte level. It is not practical to use the typical browser-based individual file downloading method to retrieve a patient cohort. To support scalable and smooth data download, we created a ruby gem called *CSR Data Inventory*, which was a software package supporting batch-downloading of CSR patient cohort. This gem supported and has been tested on multiple operating systems (Windows, Linux, and OS X; Tao, 2021).

There were five steps involved in the downloading workflow. First, the user got a data download token from the built patient cohort. Then the user can run DaT3M data downloader in the local machine and feed in the data download token. In step 3, a file list was downloaded containing the catalog of all files to be downloaded. Data downloader then traversed the file list and sent request to retrieve data files using the same data download token. In the last step, files were downloaded and stored in the user's local device. The whole workflow was highly automated. The user only needed to start the program and feed in the data download token. It was called *data slice downloader* because the user had a choice to select what data type to download during the step of data download token generation. Typically, researchers only needed specific types of data for their study, such as EEG data for signal processing and analysis or MRI data for imaging-related investigation. Slicing data can effectively reduce the workload of data download and researchers' local data management. Besides, the data downloader was robust since it supported resumable download. The first-downloaded file list contained meta-data about every file to be downloaded such as file name, path, and size. With this information, users could check if a data file was already downloaded completely. Once interrupted, the data downloader would skip those downloaded files and continue with the remaining data files.

#### 4.2.6. WaveSphere: An interactive visualization system for physiological signal recording

WaveSphere (Li, 2019) was built using the Ruby on Rails framework (ROR; Hartl, 2015; Ruby et al., 2020) and Data-Driven Documents (D3; Bostock et al., 2011). In WaveSphere, we developed the query engine based on the ROR framework for processing user requests and used D3 for building interactive interfaces for physiological signal and clinical-event annotation visualization. WaveSphere offered the following collection of main features: (1) standardized, metadata-based search and query of signal data, which provided an easy-to-use and user-friendly interface for signal data searching and retrieving based on specific user requirements; (2) online interactive and functional visualization interface for rendering waveforms, so that the signal data could be intuitively reviewed and interpreted; (3) clinical event annotation management and sharing, which provided intuitive solutions for editing and sharing cross-cohort annotation with standard and uniform annotation file formats; (4) customized signal fragments exporting and downloading function, which provided convenient ways for data exporting with complex requirements; and (5) a cross-platform, web-based applications could address the cross-platform issue with easy access by using ubiquitous Web browsers. With such an interactive system, the application provided multiple functions according to user requirements, such as query and visualizing signal fragments of interest, rendering and editing corresponding annotations, and exporting signals and annotations.

### 4.3. Risk assessment of SUDEP using MSDR

#### 4.3.1. Seizure information extraction using NLP

We used the EpSO as the core knowledge resource and constructed 4 extraction rules based on 300 randomly selected EMU discharge summaries. To evaluate the effectiveness of the extraction pipeline, we applied the constructed rules on another 200 unseen discharge summaries and compared the results against the manual evaluation of a domain expert. This pipeline was specifically designed for seizure information extraction such as the first seizure onset date, which was an important data element for SUDEP related research. Overall, our extraction pipeline achieved a precision of 0.75, recall of 0.651, and F1-score of 0.697.

#### 4.3.2. Seizure identification

To build SeizureBank, we extracted different types of signal data according to annotation files, which were created by domain experts, using our data preprocessing pipeline and then imported the analysis-ready data into the database. With such analysis-ready dataset, researchers could obtain processed data directly and spend less time on data preparation and cleaning, such as dealing with the data corruption issue, annotation labeling issue, and data extraction and segmentation from a large EDF file. Therefore, researchers could focus on seizure analysis-related tasks, such as developing statistical analysis, clustering, or other machine learning approaches.

We developed a feature-based seizure identification approach and evaluated it on three datasets, including University of Bonn seizure dataset (UBSD) including 10 subjects, the Children's Hospital Boston (CHB)-MIT scalp EEG database consisting of 23 patients, and the analysis-ready dataset in SeizureBank including 115 subjects, to construct a cross-dataset evaluation benchmark for epileptic seizure identification studies. Over 130 features, including time-domain, frequency-domain, nonlinear, and wavelet-based features, were extracted and combined with a random forest classification model to obtain the best performance on different datasets: UBSD with 99.66% F1-score and 0.9933 Kappa, CHB-MIT with 86.20% F1-score and 0.7357 Kappa, and SeizureBank dataset with 87.35% F1-score and 0.7547 Kappa (Li et al., 2019).

Seizure identification can save lives by reducing risk of SUDEP and guide treatment decisions by accurately counting seizures (Duun-Henriksen et al., 2020). Most SUDEPs occur during unsupervised times, and most commonly, the decedent is found by family or caregivers in the morning. It is also possible to be more vulnerable to SUDEP if patients with a history of seizures during unsupervised times. For example, nocturnal seizures increase SUDEP risk. During the critical interval that precedes SUDEP, an intervention may save lives (Ryvlin et al., 2013). The use of seizure identification and alerting devices in the home to notify caregivers of seizures has grown in popularity in an effort to reduce the risk of SUDEP. With the help of innovations in health technology, wearable EEG, mobile sensors, smartphones, and smart-watches, many devices and algorithms are in development. MSDR contains thousands of hours of seizure-related data from more than 2,700 patients, and it incorporates different types of seizures, such as focal seizure, generalized tonic-clonic seizure (GTC), and nocturnal seizure (seizure during sleep). Most published seizure identification algorithms were only developed and validated on smaller datasets (Li et al., 2019), and MSDR provides a large-scale dataset that can facilitate the development and validation of future seizure identification or detection algorithms.

#### 4.3.3. Seizure prediction

Our seizure prediction model was constructed using two different datasets which break the data size limitation by using a single dataset. Leveraging the large-scale labeled MSDR epilepsy dataset, we performed a real-time evaluation on a dataset consisting of over 15,840 continuing hours EEG recordings from 330 patients. Our stacked LSTM models reached 50% sensitivity, 25.43% time in warning, 13.11 min false warning per hour in average and 24.57% improvement over a random predictor, which indicated the transfer learning setup improving the seizure prediction performance using small patient-specific data (Huang, 2019).

Seizure prediction would improve quality of life and reduce disability for people with epilepsy. With seizure prediction, people could be alerted that they are likely to have a seizure within a certain number of hours, allowing them to plan and reduce their risks accordingly. It may even be possible to reduce risk by taking medication that prevents or stops seizures, or by ensuring they will be in a safe place with people who know what to do in the event of a seizure. MSDR consists of EMU data from patients with epilepsy over multiple consecutive days, meaning that the MSDR has not only seizure data, but also over terabytes of high-quality pre-ictal, inter-ictal and post-ictal data. Such continuous data provides sufficient training and testing data for the development of reliable seizure prediction algorithms.

#### 4.3.4. Post-ictal generalized EEG suppression detection

We developed a random forest-based classifier to perform PGES detection by leveraging various EEG signal features, including time-domain features, frequency-domain features, wavelet-based features, and inter-channel correlations (Li et al., 2020). Then we constructed and applied confidence-based correction rules to remove suspicious sudden changes of EEG activities. Signal features provided valuable information to characterize PGES and intermittent slow-wave brain activity. Confidence-based rules were leveraged to correct sudden changes of PGES states. In addition, we introduced a new evaluation method for assessing PGES detection results in actual clinical settings. The evaluation results on a dataset including 84 patients indicated that our method achieved a 5s-tolerance-based positive prediction rate of 95% for artifact-free EEG signals and handled the signals with different artifact levels with the rate varying from 68 to 81%.

Previous work has found an increased incidence of PGES in patients with SUDEP. The duration of PGES was directly related to the risk of SUDEP, and patients with SUDEP had longer PGES (Ryvlin et al., 2019). However, it is difficult to identify the end of PGES, even for trained clinicians. Accurate detection of the end of PGES is important for PGES characterization and SUDEP risk assessment. To date, there is no dedicated dataset for study of automatic PGES detection. Based on MSDR, we built the PGES database, which contained 116 PGES EEG recordings from 84 patients (Li et al., 2020). The volume of data in this database will continue to increase as MSDR data curation and annotation are completed. By the time the article is published, the number of patients in our PGES database increased to 174 and the number of EEG recordings increased to 268. This database will make a considerable contribution to PGES-related research.

### 4.4. Registered users and evidence of usage

Evidence of MSRD usage for scientific research included registered users in MEDCIS, research proposals submitted, and publications. There were in total 55 registered users in the current MSDR. Six projects including three R01 projects and three R21 projects had been funded by NINDS or Citizen's United for Research in Epilepsy (CURE) that adopted MSDR data for SUDEP related research (CSR, 2022). More than 60 publications, identified in the acknowledgments or references section, had appeared in scientific venues (CSR, 2022). Other user usage was reflected in the 52 saved data queries in the MEDCIS data search engine.

MSDR is not public data. But the resource is available to academic institutions and not-for-profit entities. We welcome interested research scholars to contact us for data access. We have an established standard process for reviewing and approving requests. Data use agreements and Institutional Review Boards (IRBs) are required documents.

## 5. Discussion

### 5.1. Data credibility and knowledge repositories

The quality of data and knowledge repositories is an essential and fundamental component of personalized risk assessment. High-quality data should be housed in the repository, and must be timely, accurate, clean, and unbiased, as well as stored in an appropriate format or schema (Zhang et al., 2018). The real-world data is always dirtier than what is ideal for research. It may be difficult, time-consuming, and costly to clean and reorganize data, and studies' conclusions may be affected by hidden biases. Only 20% of data scientists' time is spent building models, analyzing, visualizing, and analyzing the data, while most of their time (80%) is spent cleaning and preparing data (Patel, 2019; Li et al., 2021). However, having high-quality data is not sufficient to say the system is data-driven. Accessibility is essential; it should be joinable (able to be joined with other clinical data when needed) and shareable (a data sharing culture within the hospital eco-system so that the data can be joined). It is difficult to analyze and improve personalized risk assessment and care if clinicians/researchers do not have a coherent, accurate picture of patient flow, diagnostic processes, and complete longitudinal data acquisition processes of patients. As a final point, and very importantly, the data should be queryable and tools should be developed so that data can be sliced and diced. In order to perform assessments, large amounts of raw data should be filtered, grouped, and aggregated into smaller sets of higher-level and analysis-ready data that can assist clinicians and researchers in gaining insights into topics of interest (Li et al., 2021). Ensuring the credibility of the data and improving the knowledge base while maintaining accessibility are key issues for an effective personalized risk assessment.

Over the past 15 years, several large-scale data repositories have been established. The European Epilepsy Brain Bank was established at the University Hospital in Erlangen, Germany in 2006. Thirty-six histopathological diagnoses were collected for 2,623 children and 6,900 adults across 36 centers in 12 European countries during epilepsy surgery in this study (Blumcke et al., 2017). CSR aims to better understand the cortical, subcortical, and brainstem mechanisms responsible for SUDEP and to use a data-driven, systems biology approaches to determine whether cortical influences contribute to SUDEP. CSR consists of a curated repository of prospectively collected multimodal clinical data, the MSDR, which is linked to risk factor and outcome information for over 2,700 epilepsy patients with thousands of 24-hour recordings. MSDR data is still being continuously updated, and the increase in recruitment of patients ranges from 50 to 100 per year more recently. However, the MSDR has already been used for several ongoing studies. Our working principle is that we cannot wait to reach the ideal state of data to study SUDEP and that we will best exploit available existing data to make meaningful progress on SUDEP research.

### 5.2. Personalized risk assessment

The goal of the personalized risk assessment is to provide a heightened awareness of risk areas in clinical practice and to improve patient safety. With the rapid development of big data in medicine, the development of models for personalized risk assessment based on machine learning and artificial intelligence is now one of the hottest topics in the research field. However, most existing models and methods were built and evaluated on a small and single dataset, which makes it difficult to obtain a generally applicable and robust model (i.e., stable detection or prediction performance for different patients; Bernardi et al., 2016; Li et al., 2019, 2021).

The purpose of the evaluation is to find a better solution to make the right clinical decision to reduce labor and time costs and improve work efficiency and patient experience. However, existing evaluation techniques may not be able to reflect the performance of the model in real clinical scenarios. For example, for seizure detection, machine learning/deep learning approaches have reached performance levels that leave virtually no room for improvements (Talathi, 2017; Hussein et al., 2019; Siddiqui et al., 2020) using standard metrics include precision, recall, receiver operating characteristic (ROC) metrics, and yet few tools of practical utility have been translated to routine use in healthcare. Record-based (4 or 8 h), instead of segment-based (e.g., 10 or 60 s) evaluation approaches and time in warning measures produce poorer performance numbers but may better reflect the reality because they account for extreme imbalances of positive vs negative (e.g., 1:3,000) cases. For example, for the task of detecting post-ictal generalized electroencephalogram suppression, an important SUDEP marker, the standard evaluation method gave a spectacular F-1 measure of 0.97, but a time in warning evaluation metric dropped the F-1 score to 0.68 in our experimental study (Li et al., 2020). The challenge of class imbalance in machine learning, especially in EEG signals, lies in detecting and evaluating the effect of imbalance on the learning and testing process. EEG recordings often last for hours or days, while epilepsy events are typically brief (in minutes), resulting in extreme class imbalance. Some studies used imbalanced testing sets to evaluate the performance of their models and used traditional performance metrics. However, in practical scenarios, a model with high accuracy, sensitivity, specificity, and F-measure (over 90%) may not necessarily achieve completely satisfactory results when deployed in continuous EEG signal recording settings (the real clinical scenarios). To provide reliable data analysis, the evaluation criteria should be different according to different research tasks, for example, when detecting daily seizure events, false positives and sensitivity should be considered on an hourly scale, while seizure onset detection should focus more on the latency (in seconds). Seizure prediction, on the other hand, is more concerned with the false alarm rate with different leading time (i.e., seizure prediction horizon).

Therefore, to leverage the machine learning based approaches, or even for other types of personalized risk assessment approaches, it is important to create a new set of evaluation techniques in a more clinically relevant way based on different learning/decision tasks, and the evaluation method should have a set of evaluation metrics acceptable to clinical experts. A dataset with high patient diversity is essential to obtain a stable and reliable approach. MSDR provides a large amount of historical data of more than 2,700 patients (and growing over time) that can simulate real clinical scenarios and enhance the validation and evaluation of algorithms.

### 5.3. Future directions

Platforms such as cloud instances, which provide shared resources for emerging resources, can serve as Data Commons platforms. However, it may not be sufficient to simply expand storage capacity or add computing power to keep pace with the rapidly expanding volumes and increasingly complex nature of biomedical data. Concurrent efforts must be spent to address digital object organization challenges. We need to continue advancing research in data representation and user interfaces for human-data interaction to make our approach future-proof.

The next phase of the MSDR is the creation of a universal self-descriptive sequential (U2S) data format to represent neurophysiological data. U2S will break large, annotated, sequential data files into minimal, semantically meaningful fragments. Such fragments will be indexed, assembled, retrieved, rendered, or repackaged on-the-fly, for accommodation of distinct applications. MSDR's annotated neurophysiological data (sleep, pre-ictal, ictal, post-ictal EEG, autonomic, and respiratory measurements), containing over 6,000 seizures and 1,000 generalized convulsive seizures will be converted to the U2S format and import the resulting converted data into a new platform. This labeled time-series collection, with individual-level record-linked clinical epilepsy phenotypic data, syndromic and genetic information, biochemical, and imaging data, will support new personalized risk assessment algorithms for seizure detection, seizure prediction or forecasting, as well as SUDEP biomarker identification, with an order of magnitude increase in sample size compared to traditional studies. A new set of performance measures will be designed to reflect real-world application scenarios more faithfully, accounting for extreme biases and signal artifacts, and thus allow for more straightforward translation into practice for algorithms that perform well using such measures.

## 6. Conclusion

In this paper, we introduced MSDR, a multimodal data resource for personalized risk assessment of SUDEP, for integrating multimodal clinical data from multiple sites including seven institutions in epilepsy research. We believe that several aspects of the MSDR can help inform progress toward the implementation of future-proof multimodal clinical data integration and sharing from a domain-specific, usability-informed, bottom-up perspective.

## Data availability statement

The original contributions presented in the study are included in the article/supplementary material, further inquiries can be directed to the corresponding author/s.

## Ethics statement

The studies involving human participants were reviewed and approved by the University of Texas Health Science Center at Houston. Written informed consent to participate in this study was provided by the participants' legal guardian/next of kin.

## Author contributions

G-QZ and SL conceptualized and designed this study. LC, ST, XL, and YH implemented the tools. LC, ST, SL, and G-QZ developed the CSR metadata framework. LC, ST, XL, YH, and JH processed the data. XL wrote the manuscript with contributions from LC, ST, YH, SL, and G-QZ. All authors contributed to the article and approved the submitted version.

## Funding

This research was supported in part by the NIH through grants U01NS090408, U01NS090405, R01NS116287, and R01NS126690.

## Conflict of interest

The authors declare that the research was conducted in the absence of any commercial or financial relationships that could be construed as a potential conflict of interest.

## Publisher's note

All claims expressed in this article are solely those of the authors and do not necessarily represent those of their affiliated organizations, or those of the publisher, the editors and the reviewers. Any product that may be evaluated in this article, or claim that may be made by its manufacturer, is not guaranteed or endorsed by the publisher.

## Author disclaimer

The content is solely the responsibility of the authors and does not necessarily represent the official views of the NIH.
